# Ischemic stroke incidence in Santa Coloma de Gramenet (ISISCOG), Spain. A community-based study

**DOI:** 10.1186/1471-2377-8-5

**Published:** 2008-03-27

**Authors:** María Teresa Alzamora, Marta Sorribes, Antonio Heras, Nicolás Vila, Marisa Vicheto, Rosa Forés, José Sánchez-Ojanguren, Amparo Sancho, the "ISISCOG Study Group", Guillem Pera

**Affiliations:** 1 GPS Riu Nord-Riu Sud, Institut Català de la Salut, Major 49, 08921 Santa Coloma de Gramenet, Spain; 2 Department of Medicine, Universitat Autònoma de Barcelona, Spain; 3 Research Unit Barcelonés Nord Maresme, ICS-IDIAP Jordi Gol, Camí del Mig 36, 08303 Mataró, Spain; 4 GPS Numància, Institut Català de la Salut, Numància 23, 08029 Barcelona, Spain; 5 Unitat de Neurociències, Institut Català de la Salut, Hospital Germans Trias i Pujol, Carretera del Canyet s/n, Badalona, Spain; 6 Esperit Sant Hospital, Mossen Pons i Rabadà s/n, 08923 Santa Coloma de Gramenet, Spain; 7 GPS Can Mariné, Institut Català de la Salut, Sant Carles 79, 08921 Santa Coloma de Gramenet, Spain

## Abstract

**Background:**

In Spain, stroke is one of the major causes of death and the main cause of severe disability in people over 65 years. We analyzed the incidence of ischemic stroke, stroke subtypes, case fatality and disability at 90 days after the event in a Spanish population.

**Methods:**

A prospective community-based register of ischemic strokes was established in Santa Coloma de Gramenet (Barcelona) [116,220 inhabitants of all ages, according to the municipal census of December 31,2001], from January 1 to December 31, 2003.

Standard definitions and case finding methods were used to identify all cases in all age groups. Every patient underwent a complete clinical evaluation and systematic tests including neuroimaging (CT/MRI) and vascular studies (carotid duplex ultrasound intra and extracranial and MR angiography).

**Results:**

Over a one year period, 196 ischemic strokes were registered [107 men; median age = 76 years (range 39–98)], being the first event in 159 patients (81.1%) and a recurrent stroke in 37 (18.9%). After age-adjustment to the European population, the incidence of ischemic stroke per 100,000 inhabitants was 172 (95% CI, 148–196); 219 (176–261) in men and 133 (105–160) in women, with an annual incidence for first ischemic stroke of 139 (118–161); 165 (128–201) in men and 115 (89–140) in women. The incidence of stroke increased with age.

Stroke subtypes (TOAST classification criteria) were lacunar in 28.8%, atherothrombotic in 18.6%, cardioembolic in 26.6% and undetermined in 26.0% of patients. At 90 days, the case-fatality was 12%, and among survivors, moderate-to-severe disability was present in 45 % at 3 months.

**Conclusion:**

This prospective community-based study shows one of the lowest incidences of stroke in Europe, as well as one of the lowest case fatality and disability rates at 90 days after stroke.

## Background

In Spain, stroke is the first cause of death in women and the second in men, the main cause of severe disability in people over the age of 65 years and the second cause of dementia [1]. It has been predicted that the burden of stroke will rise in the oncoming years because of the increase in the elderly population in both the developed and developing world.

The incidence of stroke is estimated to be about 150 per 100,000 people per year in developed countries [2]. In Spain, the overall crude annual incidence of stroke was 174/100,000 and 132/100,000 inhabitants in two community studies [3,4]. The combined incidence of stroke and transient ischemic attack was 200/100,000 inhabitants [5,6].

Stroke is a disease with well-defined modifiable risk factors. Several interventions are effective in both the primary and secondary prevention of stroke and may reduce the incidence of stroke by as much as 50–80% [7,8]. However, blood pressure is well controlled in less than 20% of hypertensive patients in Spain and an important number of patients abandon hypertensive treatment within 6 months [9]. Time-trends in stroke incidence and outcome can be measured to assess the efficacy of preventive strategies and the planning of healthcare and community resources.

The present study was designed to analyze the incidence, sub-typing, case fatality and disability 90 days after ischemic stroke in an urban area of Spain using a multicenter community-based register.

## Methods

This study followed the criteria for "ideal" population-based stroke studies published by Malmgren et al. in 1987 and later updated by Bonita et al. in 1995 and Sudlow and Warlow in 1996 [10-14]. The study was approved by the regional research Ethics Committee.

### Study area and population

This prospective, population-based study was conducted in the 6 primary health care centers and 2 hospitals in Santa Coloma de Gramenet, an urban area of Barcelona, Spain, from January 1, 2003 to December 31, 2003. The study population (all residents in the community of Santa Coloma de Gramenet) comprises 116,220 inhabitants of all ages (58,115 males and 58,105 females) according to the municipal census of December 31, 2001. The population is Caucasian, of middle-low socio-economical level with a high medical advice demand and a proportion of inhabitants over 65 years of age of 15.7% (13.6% in men and 17.8% in women), similar to the Spanish proportion (17.1%) [15]. The whole population is covered by the national health service. Primary health care in this area is provided by 62 general practitioners (GPs) distributed in 6 primary health care centers. Specialized referral is provided by 2 hospitals (one tertiary university hospital and one general community hospital) which are open 24 hours a day, 7 days a week. Non residents living in this area during the ischemic event were excluded.

#### Case ascertainment

Several overlapping sources of information were used: hospitals and primary health care centers. Cases were consecutively included. To identify patients admitted to the two hospitals daily checks of hospital admission and discharge records were made by the participating neurologists (N.V. and J.S.). For precise estimation of the number of patients with non-fatal stroke managed outside the participating hospitals, the GPs in the community of Santa Coloma de Gramenet were notified of the study and regularly asked to refer all patients with stroke to the Neurology Units of the reference hospital for evaluation by the same neurologists. The authors (M.V., R.F., M.S. and M.A.) contacted GPs, nursing homes and geriatric or psychiatric long-stay institutions monthly by e-mail, facsimile or telephone calls to detect potential incident cases and to encourage early notification to the coordinating centre. In addition, a systematic search of primary care patient databases in the study area was made to seek any patient coded as stroke or acute cerebrovascular event. All patients identified were subsequently reviewed by the investigators.

Every patient with a first-ever or recurrent ischemic stroke during the study period was included as a case and was prospectively evaluated. The final diagnosis and stroke subtype classification was made by one of the two above mentioned neurologists.

### Study definitions and procedures

Diagnosis of stroke was defined according to the World Health Organization (WHO): "rapidly developing clinical signs of focal disturbance of cerebral function, lasting more than 24 hours or leading to death with no apparent cause other than that of vascular origin". This definition excludes cases of "silent stroke" detected by neuroimaging without appropriate clinical features and cases of transient ischemic attacks (TIA) (neurological deficits lasting < 24 hours). Diagnosis was based on clinical findings and CT brain imaging [16]. Stroke subtypes were classified following the TOAST criteria as lacunar, atherothrombotic, cardioembolic and undetermined [17]. Ischemic stroke was also classified into subtypes: occlusion and stenosis of pre-cerebral arteries, occlusion of cerebral arteries, transient cerebral ischemia, acute.

All events were categorized as first-ever or recurrent stroke on the basis of clinical history rather than brain imaging findings. Any stroke developing within 28 days of a previous event was considered as "progressing stroke" and was not recorded as a new event while disturbances developing ≥ 28 days were regarded as a recurrent event [18]. "Attack rate" includes all stroke events (first-ever and recurrent stroke), whereas "incidence rate" describes first-ever stroke [18]. A first-ever stroke in a patient with a previous transient ischemic attack was coded as incident and a new stroke in a patient with a previous stroke was coded as recurrent. Neither new cases of "transient ischemic attack" nor intracerebral hemorrhages were included in this study.

#### Patient assessment

A combination of "hot pursuit" (prospective screening of hospital admissions and referrals from GPs, nursing homes and long-stay institutions) and "cold pursuit" (retrospective identification of potential cases from hospitals and Primary Health Centers medical records) was used to determine patients with stroke.

Before patient registry, surviving patients received an information letter and provided written consent to for inclusion in the registry and to undergo follow-up. Family consent (next of kin) was obtained from patients who were severely ill or unconscious. All patients with suspected stroke were transferred to one of the two participating hospitals and examined by the consulting neurologists. Data on previous therapy, vascular risk factors and medical history were collected from medical records. Systematic investigations included neurological examination, 12-lead ECG, chest radiography, and routine blood tests including fasting glucose, cholesterol concentrations, and complete blood analysis. Non contrast brain CT scan was performed immediately after hospital admission in all patients. Control CT or MRI was performed during the first two weeks of hospitalization to confirm the ischemic lesion. On identification of a case, data was manually introduced in a computer database designed for this study.

Patients were followed by GPs until 3 months after stroke onset. Case fatality was defined as the proportion of registered patients with stroke who died within 90 days of the stroke. The cause of death was established by the GP certifying death at home or was determined from all available medical records on death in the hospital or a nursing-home institution. There was no need to review death certificates since all were signed by the GPs or neurologists (in-hospital death) participating in the study. Disability at 90 days was measured with the Barthel Index (BI) of daily living activities [19]. The BI was evaluated by nurses who attended patients at the primary health care centers, patient homes or institutions, and classified as severe (< 60), moderate (60 to 85) and mild or no disability (90 to 100). The BI was not recorded in two patients who were alive at the end of the follow-up.

### Statistical analysis

Incidence rates and 95% confidence intervals (CI) were calculated per 100,000 inhabitants. The denominator for the calculation of the incidence rate was the updated census data from the 2001 (116,202 inhabitants). Incidence rates were calculated for the whole population and by sex in the following age groups: < 45 years, 45 to 49, 50 to 54, 55 to 59, 60 to 64, 65 to 69, 70 to 74, 75 to 79, 80 to 84 and >=85 years. For comparison with other community-based studies, the rates were adjusted to the 2001 European population [20].

Sex and age-specific stroke attack rates were calculated. To compute incidence rates and their confidence intervals (row and standardised) we used the statistical package: CIA v1.0 (Professor MJ Gardner & BMJ, 1989). All confidence intervals are at level of 95%.

## Results

From January 1st to December 31st, 2003, 247 patients with a suspected acute cerebrovascular event were identified. Most strokes were admitted to one of the two participating hospitals (92%), but a few cases to nursing homes or long-stay institutions (1 %), were reported directly by primary health care center physicians (6%) or were admitted to other hospitals outside the community (1%). Screening of death certificates was not performed. After comprehensive evaluation in all patients ischemic stroke was diagnosed in 196 cases; 159 patients (81.1%) with a first-ever ischemic stroke and 37 (18.9%) a recurrent stroke. 51 patients were excluded because of intracerebral hemorrhage (n = 50) or brain tumor (n = 1), thus, a total of 196 patients with cerebral infarction were registered.

The cases included 107 males (55%) and 89 females (45%) with a mean age of 74 years (71 for men and 78 for women) and median age was 76 years (range 39 to 98). Among cases, prevalences of several known risk factors were: prior TIA (6%), hypertension (66%), dyslipidemia (31%), current smoker (16%), diabetes mellitus (34%), peripheral arterial disease (6%), atrial fibrillation (26%), angina (10%), myocardial infarction (11%), and sleep apnea (5%).

The global incidence for ischemic stroke was 169/100,000 (95% CI, 145–192), and the crude annual incidence for first-ever ischemic stroke was 137/100,000 (95% CI, 116–159). The age-specific annual attack and incidence rates of ischemic stroke are shown in tables 1 and 2, respectively. After age-adjustment to the 2001 European population, the incidence for ischemic stroke was 172 (95% CI, 148–196) per 100,000 inhabitants; 219 (176–261) in males and 133 (105–160) in females and the annual incidence for first-ever ischemic stroke was 139 (95% CI, 118–161) per 100,000 inhabitants; 165 (128–201) in males and 115 (89–140) in females. The incidence rate increased with age, with 4 cases per 100,000 individuals in subjects under 45 years of age, 58 in those from 45–49 years, 135 in the 50–54 year-old group, 105 from 55–59 years, 271 from 60–64 years, 276 from 65–69 years, 788 from 70–74 years, 1112 from 75–79 years, 1338 from 80–84 years, and 1610 in subjects ≥ 85 years. This increase in incidence with age was observed in both sexes, with a higher incidente in males in all age groups.

**Table 1:** Age- and sex-specific attack rates of ischemic stroke per 100,000 population of Santa Coloma de Gramenet, Spain, 2003.

**Age **(years)	**Men**		**Women**		**Total**	
	
	Events/at risk	Rate (95% CI)	Events/at risk	Rate (95% CI)	Events/at risk	Rate (95% CI)
< 45	3/35418	8 (0–18)	0/32375	-	3/67793	4 (0–9)
45–49	2/3350	60 (6–214)	2/3489	57 (6–201)	4/6839	59 (1–116)
50–54	8/3816	210 (92–409)	3/4340	69 (0–147)	11/8156	135 (55–215)
55–59	8/4186	191 (59–323)	1/4369	23 (0–68)	9/8555	105 (37–174)
60–64	14/3445	406 (226–677)	4/3197	125 (31–323)	18/6642	271 (146–396)
65–69	9/3074	293 (128–555)	8/3081	260 (116–507)	17/6155	276 (145–407)
70–74	23/2129	1080 (690–1620)	13/2440	533 (287–909)	36/4569	788 (532–1040)
75–79	18/1381	1300 (775–2060)	20/2037	982 (604–1510)	38/3418	1110 (787–1520)
80–84	12/773	1550 (800–2690)	17/1394	1220 (714–1950)	29/2167	1340 (897–1910)
≥ 85	10/543	1840 (885–3360)	21/1383	1520 (946–2310)	31/1926	1610 (1090–2280)

**Row/Crude**	107/58115	**184 (149–219)**	89/58105	**153 (121–185)**	196/116220	**169 (145–192)**
**Standardised (Europe)**		**219 (176–261)**		**133 (105–160)**		**172 (148–196)**

CI = Confidence Interval

**Table 2:** Age- and sex-specific annual incidence rates of first-ever ischemic stroke per 100,000 population of Santa Coloma de Gramenet, Spain, 2003.

**Age **(years)	**Men**		**Women**		**Total**	
	
	Events/at risk	Rate (95% CI)	Events/at risk	Rate (95% CI)	Events/at risk	Rate (95% CI)
< 45	3/35418	8 (0–18)	0/32375	-	3/67793	4 (0–9)
45–49	2/3350	60 (6–214)	1/3488	29 (6–165)	3/6838	44 (0–93)
50–54	7/3815	183 (79–372)	2/4339	46 (0–110)	9/8154	110 (38–182)
55–59	6/4184	143 (29–258)	1/4369	23 (0–68)	7/8553	82 (21–142)
60–64	13/3444	377 (201–641)	4/3197	125 (30–323)	17/6641	256 (134–378)
65–69	6/3071	195 (67–421)	8/3081	260 (116–507)	14/6152	228 (108–347)
70–74	17/2123	801 (470–1280)	11/2438	451 (226–812)	28/4561	614 (387–841)
75–79	13/1376	945 (507–1610)	17/2034	836 (482–1340)	30/3410	880 (592–1250)
80–84	9/770	1170 (531–2200)	15/1392	1080 (604–1780)	24/2162	1110 (714–1640)
≥ 85	6/539	1110 (409–2410)	18/1380	1300 (775–2060)	24/1919	1250 (800–1850)

**Row/Crude**	82/58090	**141 (111–172)**	77/58093	**133 (103–162)**	159/116183	**137 (116–158)**
**Standardised (Europe)**		**165 (128–201)**		**115 (89–140)**		**139 (118–161)**

CI = Confidence Interval

A brain CT scan was performed immediately after hospital admission in all patients. Control CT scan was performed during the first two weeks of hospitalization in 110 patients (56%), and MRI in 90 patients (46%). Other examinations included 12-lead ECG in 192 patients (98%), transthoracic echocardiography in 86 patients (44%), carotid Dupplex ultrasound intra and extracranial in 159 patients (81%) and MR angiography in 9 patients who also underwent the ultrasound study. According to the TOAST classification, 37 (18.6%) patients presented a large artery atherothrombotic stroke, 52 (26.6%) a cardioembolic stroke, 56 (28.8%) a lacunar stroke and 51 (26.0%) a stroke of undetermined origin.

Two patients (1.26%) were lost to follow up within the first 90 days after the event. Among survivors at 3 months, severe disability was present in 21.9% (95% IC:15.4–28.5), moderate disability in 22.6% (95% IC:16.0–29.2) and mild or non-disability in 55.5% (95% IC:47.7–63.3). Severe disability increased with age, from 1.16% in people younger than 50 up to 8.73% in people with 75–85 years. However, this trend was broken for people older than 85 (5.24%). The same trend was found for moderate disability: 0.58% among younger than 50 up to 19.11% among people 75–85 years old, 2.33% in people older than 85.

The case fatality at 90 days was 12% (95% CI: 7–17), being higher in women (60% of total cases). We found the following mortality rates at 90 days by age: 15% (< 65 years), 10% (66–75), 45% (76–85) and 30% (> 85). 50% of death occurred in the first month, 40% at the second month and 10% at the third month.

## Discussion

To our knowledge, this is the first community-based study on ischemic stroke in Spain using "hot pursuit" and "cold pursuit", in a geographic area with a large, stable, well-defined population and a close relationship between hospital neurology departments and GPs using standard and accepted tests for the diagnosis of stroke in all patients. Prior stroke incidence in a Spanish population was estimated at between 163 and 257 cases per 100,000 inhabitants with a crude annual incidence rate for first-ever stroke from 132 and 174 cases [3,5], although these studies did not follow the "ideal" criteria proposed by Malmgrem, Bonita, Sudlow and Warlow [10-13]. Our study shows a crude annual incidence rate for first-ever ischemic stroke of 137 cases per 100,000 inhabitants, being one of the lowest reported in community-based studies using similar methodology, even when standardized to the European population (table 3).

**Table 3:** Annual incidence rate cerebral infarction and adjusted annual incidence rate ischaemic stroke.

**Study**	**Oxford Community Stroke Project**	**MONICA**	**Perth Community Stroke Study**	**ARIC**	**INNHERRED**	**ERLANGEN**	**L'AQUILA**	**South London Stroke Register**	**Oxford Vascular Study**	**ISISCOG**
**Year**	1981–84	1985–87	1989–90	1987–95	1994–96	1994–96	1994–98	1995–98	2002–04	2003
**Place**	UK	Europe & China	Australia	USA	Norway	Germany	Italy	UK	UK	Spain
**n **(population)	86,487	2,900,000	138,704	15,792	70,000	101,450	297,838	234,533	90,542	116,202
**Age **(years)	No age restriction	35–64	No age restriction	45–64	≥ 15	No age restriction	No age restriction	-	No age restriction	No age restriction
**Mean Age **(years)	67.4	-	72.7	-	-	-	74.8	71.7	74.1	74
**Overall Stroke **(for 100,000)	227	101–285 men 47–198 women	258-	-	312 285 men 338 women-	-	275	-	162	-
**Incidence rate cerebral infarction **(for 100,000)	193	-	178 189 men 166 women-	-	232	119 men 154 women	221 men 220 women	-	142	137 141 men 133 women
**Adjusted Incidence rate ischemic stroke **(for 100,000)	-	-	World 132 men 77 women	180 women	-	European 134	European 228	European128 World 83	-	European 139 World 65
**CT based diagnoses **(%)	98	-	-	CT scan: 84 MRI: 27	87.5	95	89	CT/MRI: 88.3	-	100
**30 day case fatality **(%)	17.8	30	22	7.6	19.2	19.4	25.6	25.7	17.2	-
**90 day case fatality **(%)	.	-	-	-	-	28.5	-	32.9	-	12

Arterial hipertensión was the most prevalent cardiovascular risk factor in these patients similar to the results of most studies [14].

Determination of the incidence of ischemic stroke in our area may be of interest to monitor possible differences in different European regions. Another point of note is that the probability of having a stroke is normally calculated with the Framingham equation. However, the REGICOR study carried out in Spain [21] has demonstrated that this equation, estimated in the American population, overestimates the risk of vascular events in European populations, especially in the Mediterranean peoples. Thus, the real incidence of stroke in our country may be lower than that of other European countries. Moreover, this study will be useful to monitor the evolution of incidence, case fatality and disability of our population since, after having undertaken this study, an Institutional Plan has been implemented for protocolized and emergency care to patients with stroke. This will undoubtedly have a positive influence on the evoluation of this disease as has been demonstrated in other studies published in the literature [14].

Due to the increasing age of the general population in our country, it is important to obtain information in old and very old patients (> 84 years old), in order to provide adequate health care services and to plan socioeconomic resources. Accordingly, was not restricted to any group of age. In fact, 16% of all strokes were observed in patients over the age of 84 years, representing 1.7% of the population in our area [3,18,22].

In this study only 8% of patients were not-admitted to hospital in the acute phase of stroke, a proportion similar to that observed in many other developed countries, especially in urban areas [23-35]. This is in accordance with the Helsinborg Statement that defines stroke as an emergency and points out the need for all patients to be rapidly hospitalized in specialized care units after the onset of symptoms [30,31,34].

The present study shows a mortality rate lower than that reported in prior population-based studies [3-5]. Although several factors not assessed in the present study may explain these differences, mortality and dependency have been reduced in recent because of improvement in the general management and care of acute stroke patients [32-34]. An important effort has been made to avoid underestimation of stroke incidence, combining "hot and cold pursuit" and contacting all the GPs, nursing homes and long-stay institutions in addition to an exhaustive scan of medical and admission records. Correct case ascertainment has been achieved using 2 expert neurologists and CT scan in all cases.

Diagnosis was based on clinical findings and CT brain imaging. A CT scan was performed in 100% of the cases, and a control CT scan was performed in the 56% of patients. Magnetic resonance imaging was carried out in 46%. On suspicion of different subtypes of stroke, an MRI is more indicated than a CT scan. In 2 % of the cases both imaging tests were required.

Comparison between this and other epidemiological studies [3-5] carried out in our country is difficult due to differences in the time periods studied (in the last decades confirmation of diagnosis with neuroimaging techniques has varied markedly), as well as in methodologies implemented. In the ISISCOG study, cranial CT was used in 100 % of the cases while cerebral CT was only performed in 70 % of the cases studied by Caicoya et al. [4], in 72 % of the patients included by Jover et al. [5] and the percentage of cranial CT carried out in the study by Lopez Pousa et al. was not reported [3]. In this latter study only 35 % of the patients with stroke were hospitalized which may negatively influence the functional prognosis and mortality of these patients. In the study by Jover et al. 62.6 % of the patients were admitted to hospital, although this was not a populational study since only patients attended in the emergency department were studied [5]. The different percentages of hospital admissions have varied over time in our country, which may reflect both health care education of the patient and that of the professional as well as the policy of patient admission at a hospitalary level in the different decades.

Stroke subtypes according to the TOAST classification were similar to those observed in other European countries [23,25,26]. As expected, the proportion of lacunar stroke subtype was lower in the ISISCOG study than in the ARIC study due to differences in the prevalence of hypertension and hypertension control in Europe and the United States [33,36].

The proportion of recurrent strokes (18.4%) during the study period was lower than in the Erlanger study (23.0%) in Germany [26] and the Innherred study (27.2%) in Norway [29].

The case fatality at 90 days was 12% (95% CI: 7–17), being higher in females (60% of total cases). We believe data collection on case fatality and disability to be correctly registered, despite not having reviewed the death certificates since autopsies are not routinely undertaken in all deaths in Spain and the death certificates were signed by the GPs or neurologists (in hospital deaths) participating in the study. Moreover, we determined the number of survivors at 90 days with the follow up using the Barthel Index.

Among survivors at 3 months, severe disability was present in 21.9%, moderate disability in 22.6% and mild or non-disability in 55.5%. Two patients were lost to follow up among the survivors in the first 90 days. All survivors (except the latter two) performed the Barthel test to determine the grade of dependence by telephone interview or personally if the patient was unable to respond by telephone. The loss to follow up was due to the transfer of the patients to other provinces in which the patients could not be reached by telephone to perform the Barthel Index.

Case fatality and disability may have been influenced by the possibility of receiving specialized health care early since the population studied was an urban population located with 20 minutes from an acute care hospital with a neurology department.

The first limitation of the study may be the exclusive collection of ischemic stroke. Another limitation may be the diferent sources of the information on the denominator and the numerator. When a stroke case is detected and the clinical history reveals that this person had a stroke before the study started, this would no longer be considered a first stroke. However, this person should also be excluded from the denominator, because he/she was not at risk of first stroke at the time of the initiation of the study. This could artificially make stroke rates lower than they actually are, for instance, in comparison to follow-up studies in which information from the population and the outcome comes from the same source. Nonetheless, we believe that this is not a large problem and probably has only a limited impact on the results.

## Conclusion

In summary, this prospective community-based study shows a lower age-adjusted incidence of stroke in northeastern Spain than other European studies and a lower case-fatality at 90 days after stroke. These findings have established the bases in order to determine changes in stroke incidence in the next years. Recently a new acute stroke unit has been implemented in our area, just after this study was carried out. Studies of time-trends in stroke incidence are required to ascertain whether the implementation of primary prevention strategies and an acute stroke unit in our area are associated with a potential fall in stroke incidence, disability and case fatality.

## Competing interests

The author(s) declare that they have no competing interests.

## Authors' contributions

MTA, NV, MS, MV, and RF participated in the design of the study; MTA, MS, JS and AS contributed to the coordination study; GP participated in the statistical calculations. All the authors have read and approved the final manuscript.

## Pre-publication history

The pre-publication history for this paper can be accessed here:


